# Decreased Neurofilament L Chain Levels in Cerebrospinal Fluid and Tolerogenic Plasmacytoid Dendritic Cells in Natalizumab-Treated Multiple Sclerosis Patients – Brief Research Report

**DOI:** 10.3389/fncel.2021.705618

**Published:** 2021-07-26

**Authors:** Adriel S. Moraes, Vinicius O. Boldrini, Alliny C. Dionete, Marilia D. Andrade, Ana Leda F. Longhini, Irene Santos, Amanda D. R. Lima, Veronica A. P. G. Silva, Rafael P. C. Dias Carneiro, Raphael P. S. Quintiliano, Breno B. Ferrari, Alfredo Damasceno, Fernando Pradella, Alessandro S. Farias, Charles P. Tilbery, Renan B. Domingues, Carlos Senne, Gustavo B. P. Fernandes, Felipe von Glehn, Carlos Otavio Brandão, Carla R. A. V. Stella, Leonilda M. B. Santos

**Affiliations:** ^1^Neuroimmunology Unit, Department of Genetics, Evolution, Microbiology, and Immunology, University of Campinas (UNICAMP), Campinas, Brazil; ^2^Department of Immunology and Rheumatology, University of Alabama at Birmingham, Birmingham, AL, United States; ^3^Department of Neurology, University of Campinas, Campinas, Brazil; ^4^MS Clinic of Santa Casa de São Paulo (CATEM), São Paulo, Brazil; ^5^National Institute of Science and Technology on Neuroimmunomodulation (INCT-NIM), Oswaldo Cruz Institute, Oswaldo Cruz Foundation, Rio de Janeiro, Brazil; ^6^Senne Liquor Diagnóstico, São Paulo, Brazil; ^7^Hospital Israelita Albert Einstein, São Paulo, Brazil

**Keywords:** neuroinflammation, HLA-DR, HLA-G, PD-L1, neurofilament light chain, cerebrospinal fluid

## Abstract

**Background:**

Neurofilament Light (NfL) chain levels in both cerebrospinal fluid (CSF) and serum have been correlated with the reduction of axonal damage in multiple sclerosis (MS) patients treated with Natalizumab (NTZ). However, little is known about the function of plasmacytoid cells in NTZ-treated MS patients.

**Objective:**

To evaluate CSF NfL, serum levels of soluble-HLA-G (sHLA-G), and eventual tolerogenic behavior of plasmacytoid dendritic cells (pDCs) in MS patients during NTZ treatment.

**Methods:**

CSF NfL and serum sHLA-G levels were measured using an ELISA assay, while pDCs (BDCA-2^+^) were accessed through flow cytometry analyses.

**Results:**

CSF levels of NfL were significantly reduced during NTZ treatment, while the serum levels of sHLA-G were increased. Moreover, NTZ treatment enhanced tolerogenic (HLA-G^+^, CD274^+^, and HLA-DR^+^) molecules and migratory (CCR7^+^) functions of pDCs in the peripheral blood.

**Conclusion:**

These findings suggest that NTZ stimulates the production of molecules with immunoregulatory function such as HLA-G and CD274 programmed death-ligand 1 (PD-L1) which may contribute to the reduction of axonal damage represented by the decrease of NfL levels in patients with MS.

## Introduction

Natalizumab (NTZ) is one of the most effective therapy for relapsing-remitting multiple sclerosis (RRMS). Its most known mechanism of action is the blockage of α4-integrins (CD49d), thus preventing the entry of autoreactive T- and B- lymphocytes in the central nervous system (CNS) (Bielekova and Becker, 2010). Indeed, increasing evidence points to the restraining of auto-aggressive T- and B- lymphocytes in the peripheral blood of NTZ-treated MS patients (Kimura et al., 2016; Saraste et al., 2016; Bühler et al., 2017; Traub et al., 2019). At the same time, reduced CNS inflammatory activity associated with lower levels of neurofilament light chain (NfL) in the cerebrospinal fluid (CSF) has been described during NTZ treatment (Gunnarsson et al., 2011; Romme Christensen et al., 2014). Despite targeting auto-aggressive lymphocytes, it is known that NTZ may also impact other subsets, such as dendritic cells (DCs). However, few studies have explored this aspect (Kivisäkk et al., 2014). Even more scarce are reports investigating possible tolerogenic functions induced by NTZ in subsets of dendritic cells.

We have investigated the CSF levels of NfL in NTZ-treated MS patients and we have simultaneously demonstrated that plasmacytoid dendritic cells (pDCs) exhibit tolerogenic molecules such as HLA-G^+^ and CD274^+^ [also named programmed death-ligand 1 (*PD-L1*)] either in peripheral blood or in CSF during this therapy.

## Methods

### Ethics Statement

Patients were recruited during regular follow-up visits at the University of Campinas (UNICAMP) Hospital, São Paulo, Brazil. Peripheral blood (PB) and CSF from MS patients and healthy donors were collected after they agreed to participate and signed the Term of Consent. The research was approved by the University of Campinas Committee for Ethical Research (number 26291).

### Patients

Thirty-three patients (mean age 32.1 ± 9.3 years) with clinically diagnosed RRMS according to revised McDonald criteria (Polman et al., 2011) and twenty healthy individuals were included in the study (Table 1). Among MS patients, seventeen were under NTZ therapy and sixteen were untreated. MS patients consented to donate excess CSF and blood during routine lumbar puncture.

**TABLE 1:** Clinical characteristics of MS cohort.

	*Subjects*	*Age (years, median)*	*Gender ♀:♂*	*Oligoclonal bands (OCBs)* (±)
NTZ-RRMS patients	17	35,0 (21–49)	13/4	7/6*
Untreated-RRMS patients	16	37,5 (18–57)	13/3	12/4
Healthy donors	20	32,5 (20–45)	14/6	–

*Not all patients were tested for OCBs.

### Quantification of Neurofilament-Light Chain (NfL) in the Cerebrospinal Fluid (CSF)

Cerebrospinal fluid was obtained through a lumbar puncture and was centrifuged for 10 min at 200 × *g* and stored at −80°C. Neurofilament light chain (NfL) levels in the CSF were determined from investigated MS patients and healthy donors using a solid-phase sandwich ELISA. The method was developed in duplicate according to the manufacturer’s (Uman Diagnostics, Sweden) instructions. The standard curve ranged from 100 to 10,000 pg/mL.

### Quantification of Soluble HLA-G in the Serum

HLA-G levels were determined in the serum from MS patients and healthy donors using a solid-phase sandwich ELISA. This methodology specifically detects soluble HLA-G1 and G5 in a β-microglobulin (β2m)-associated form and was executed according to the manufacturer’s (EXBIO Praha, Czechia) instructions.

### Flow Cytometry Analyses (FACS)

Peripheral blood mononuclear cells (PBMC) were obtained by Ficoll-Hypaque^®^ gradient. pDCs were identified based on their selective expression of surface antigen CD303^+^, named blood DC antigen 2 (BDCA-2^+^) (Peng et al., 2020). Anti-human antibodies: BDC2-APC, HLA-DR APC-Cy7, CD274 PE-Cy7, HLA-G PerCP5.5, and CCR7 PE were obtained from BD Bioscience (United States). Data samples were acquired using Gallios (Beckman Coulter) flow cytometer and analyzed using BD FACSDiva software (BD Biosciences, United States).

### Statistical Analysis

Statistical analysis was determined using a non-parametric test (Kruskal–Wallis). Dunn’s multiple comparison test was used as *post hoc* of Kruskal–Wallis. Grubbs’s test was performed to exclude outliers. Differences were considered statistically significant with a *p* < 0.05, *p* < 0.01, and *p* < 0.001 values. Results are expressed as mean and standard deviation.

## Results and Discussion

**FIGURE 1:**
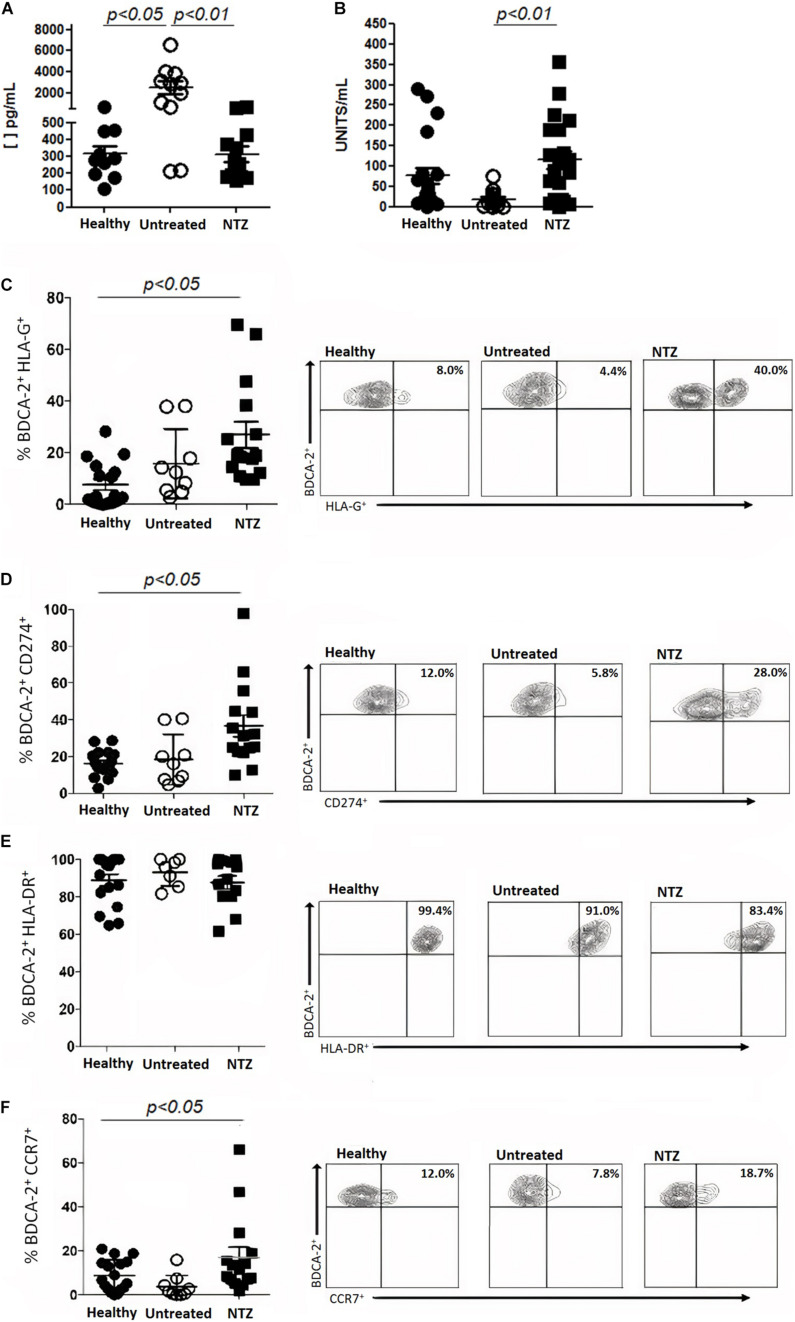
Neurofilament L chain (NfL), soluble HLA-G (sHLA-G) and tolerogenic properties of plasmacytoid dendritic cells (pDCs) during Natalizumab. **(A)** NfL levels were measured in the cerebrospinal fluid and **(B)** sHLA-G levels were measured in the serum, both obtained from MS patients and healthy donors. **(C)** HLA-G^+^, **(D)** CD274^+^, **(E)** HLA-DR^+^, and **(F)** CCR7^+^ expressed by pDCs (BDCA-2^+^) from healthy donors, untreated and NTZ-treated MS patients were evaluated using flow cytometry analyses. Results are represented by mean and standard deviation.

In the last few years, there has been an attempt to establish accessible biomarkers for measuring therapeutic effectiveness or even for predicting clinical outcomes for MS patients. NfL has emerged as the major studied CSF biomarker for this purpose (Piehl et al., 2018; Dalla Costa et al., 2019; Damasceno et al., 2019; Sellebjerg et al., 2019; Delcoigne et al., 2020). Moreover, NTZ decreased CSF NfL levels are associated with both clinical and MRI improvement of MS patients (Gunnarsson et al., 2011). Thus, confirming previous reports, we show here that NTZ treatment decreases NfL levels to similar amounts found in the CSF from control group (Figure 1A). These finding reinforces the importance of NfL as an effective marker for assessing therapy effectiveness. In line with this and considering the importance of establishing accessible serum markers for managing MS treatment, we demonstrated an increase of sHLA-G in the serum from NTZ-treated patients (Figure 1B). Increased levels of sHLA-G in the CSF were found particularly in inactive MRI-lesions from MS patients (Fainardi et al., 2003), suggesting that immunosuppressive properties of sHLA-G may down-modulate auto-aggressive CD8+ and CD4+ T cells during disease. Thus, the results suggested that in NTZ-treated patients, despite the entrapment of auto-aggressive cells in the peripheral blood, as its main mechanism of action, immunoregulatory functions may also emerge during the therapy.

A previous study demonstrated that mRNA expression of *FoxP3* is downregulated in CD49d + CD4 + T lymphocytes from NTZ-treated MS patients, suggesting a disrupted balance between inflammatory and regulatory functions for T lymphocytes during this therapy (Kimura et al., 2016). Thus, we investigated whether tolerogenic behavior may be exerted by other cell subsets such as DCs. Specifically, pDCs are known as a major producer of type I IFNs and exert either pro- or anti-inflammatory functions. Indeed, pDCs were previously demonstrated in inflamed meninges and in active MS lesions (Lande et al., 2008). Moreover, our group described increased number of pDCs in the CSF from relapsing MS patients (Longhini et al., 2011). Here, we show that NTZ-treated MS patients exhibit, in the peripheral blood, increased expression of immunoregulatory molecules such as HLA-G^+^ (Figure 1C) and CD274^+^ (Figure 1D). Since pDCs reach the CNS, we can rise the hypothesis that this subset may promote the expansion of myelin-specific regulatory T cells through interaction of myelin-Ag with MHC II (HLA-DR^+^) (Figure 1E) as previously shown in MS experimental model (Irla et al., 2010). Moreover, CD274^+^ (Figure 1D) expressed on surface of dendritic cells would also dampen auto-aggressive responses of auto-reactive T lymphocytes (Peng et al., 2020). Confirming its migratory capacities, we showed that pDCs also exhibit up-regulation of chemokine CCL19 receptor (CCR7^+^) expression (Figure 1F). In agreement with these observations, it was previous demonstrated the increased amounts of chemokine CCL19 in the CSF from MS patients, particularly during relapses (Krumbholz et al., 2007).

Thus, pDCs expressing immunoregulatory molecules in peripheral blood from NTZ-treated MS patients, in association with normal levels of NfL in CSF, seem to indicate features of good treatment response. Further investigations about tolerogenic functions by pDCs during NTZ may reveal important and yet unknown immunomodulatory mechanisms promoted by this therapy. Moreover, beyond NfL, the discovery of novel accessible soluble and cellular biomarkers is mandatory for the better clinical and therapeutic interventions in the future of MS clinical practice.

## Data Availability Statement

The raw data supporting the conclusions of this article will be made available by the authors, without undue reservation.

## Ethics Statement

The studies involving human participants were reviewed and approved by the UNICAMP Committee for Ethical Research (process number: 26291). The patients/participants provided their written informed consent to participate in this study.

## Author Contributions

AM and VB performed most of the experiments. ADL, VS, RQ, BF, FP, MA, ALL, and IS performed soluble HLA-g assay. ACD performed flow cytometry experiments. AF analyzed the data. RPD, AD, CT, RBD, CS, GF, FG, CB, and CRS diagnosed, selected the volunteers, collected the samples, and organized the clinical data. LS designed and coordinated the study. AM, VB, and LS wrote the manuscript with inputs from co-authors. All authors contributed to the article and approved the submitted version.

## Conflict of Interest

LS received a research grant from BIOGEN and a consultation honorarium from BIOGEN and ROCHE. The remaining authors declare that the research was conducted in the absence of any commercial or financial relationships that could be construed as a potential conflict of interest.

## Publisher’s Note

All claims expressed in this article are solely those of the authors and do not necessarily represent those of their affiliated organizations, or those of the publisher, the editors and the reviewers. Any product that may be evaluated in this article, or claim that may be made by its manufacturer, is not guaranteed or endorsed by the publisher.
